# Controlling Soil-Transmitted Helminthiasis in Pre-School-Age Children through Preventive Chemotherapy

**DOI:** 10.1371/journal.pntd.0000126

**Published:** 2008-03-26

**Authors:** Marco Albonico, Henrietta Allen, Lester Chitsulo, Dirk Engels, Albis-Francesco Gabrielli, Lorenzo Savioli

**Affiliations:** 1 Ivo de Carneri Foundation (IdCF), Milano, Italy; 2 Department of Control of Neglected Tropical Diseases, World Health Organization, Geneva, Switzerland; London School of Hygiene & Tropical Medicine, United Kingdom

## Abstract

Pre-school age children account for 10%–20% of the 2 billion people worldwide who are infected with soil-transmitted helminths (STHs): *Ascaris lumbricoides* (roundworm), *Trichuris trichiura* (whipworm), and *Ancylostoma duodenale/Necator americanus* (hookworms). Through a systematic review of the published literature and using information collated at World Health Organization headquarters, this paper summarizes the available evidence to support the recommendation that pre-school children should be included in regular deworming programmes. The first section describes the burden of STH disease in this age group, followed by a summary of how infection impacts iron status, growth, vitamin A status, and cognitive development and how STHs may exacerbate other high mortality infections. The second section explores the safety of the drugs themselves, given alone or co-administered, drug efficacy, and the importance of safe administration. The third section provides country-based evidence to demonstrate improved health outcomes after STH treatment. The final section provides country experiences in scaling up coverage of pre-school children by using other large scale public health interventions, including vitamin A programmes, immunization campaigns, and Child Health days. The paper concludes with a number of open research questions and a summary of some of the operational challenges that still need to be addressed.

## Introduction

It is well known that pre-school-age children (PSAC) pay the highest toll of mortality and morbidity for the “high profile” diseases: it is estimated that more than 10 million children die every year from malaria, acute respiratory infections, diarrhoea, HIV/AIDS, vaccine-preventable diseases (including measles and tetanus), and from malnutrition and neonatal complications before reaching their 5th birthday [1]. Young children also pay heavily for some of the less well-known diseases, including infection with one or more of the soil-transmitted helminths (STHs): *Ascaris lumbricoides* (roundworm), *Trichuris trichiura* (whipworm), and *Ancylostoma duodenale/Necator americanus* (hookworms).

PSAC comprise between 10% and 20% of the 3.5 billion people living in STH-endemic areas [2], and although these infections are not among the big killers, they endanger children's health in a subtle and debilitating way. Chronic infections compromise healthy growth, cognitive development, physical fitness, and iron status, and affect the immune response of infected children.

Growth faltering typically occurs between 6 months and 2 years of age, overlapping with the time when STH infections begin to establish themselves. Globally it is estimated that almost 200 million PSAC are stunted, 33% of whom live in developing countries; STH infections are an important factor contributing to malnutrition in this age group [3].

From the health perspective, there is now ample evidence clearly demonstrating that regular treatment of helminth infections produces immediate as well as long-term benefits, significantly contributing to the development of affected individuals, particularly children [4]–[6]; from the operational perspective, several issues have also strengthened the argument for including anthelminthic drugs in large scale public health interventions: the drugs are safe and simple to administer, although training for the drug administrators is essential, particularly when very young children are being targeted [7],[8]. Anthelminthic drugs are popular with affected communities, which boosts compliance and therefore increases the overall coverage of interventions. STH treatment is also one of the key components of the preventive chemotherapy package concept [9]. With regard to the costs and benefits of STH deworming, the best evidence relates to school-age children (SAC) since they are easy to reach en masse through the school system, making it a low-cost intervention, while treatment of this group produces substantial returns in terms of reduced morbidity, improved growth, and improved educational outcomes [10]. The same economies of scale are likely to exist for younger children who are increasingly being reached through large scale interventions, including vitamin A campaigns, vaccination programmes, and Child Health Days [2],[11].

In sum, recent recommendations encourage governments and policy makers to invest in helminth control as an asset for development [6]. Like any public health intervention, however, deworming for STH infections must be justified by evidence and judiciously implemented, especially when very young children are targeted for treatment.

This review summarizes state-of-the-art published data and information collected through WHO's Global Database on STHs on the evidence behind treating PSAC for STH infections. Since there is no consistent definition of PSAC in the medical literature in terms of age and/or school enrolment, we have used age as the key defining indicator in this review; thus, any child between 1 and 5 years inclusive (i.e., between the 1st and the 6th birthday) was considered a PSAC irrespective of his/her school enrolment status. Children in their first year of life are considered as infants and children from their 6th birthday up to the age of 15 years inclusive (i.e., up to their 16th birthday) are considered to be school-age children.

## Burden of Disease Associated with STH Infections in PSAC

### Numbers Infected

STH infections are widespread globally. As the prevalence and intensity of infection peaks in school age, SAC have traditionally been the priority target group for treatment. Nevertheless, as soon as an infant starts to explore its environment, thus coming into contact with contaminated soil, he/she is at risk of infection according to the levels of transmission in the area.

Recent estimates indicate that more then 2 billion people are infected with STHs worldwide, a significant proportion of which are PSAC (Table 1). A selection of studies that have reported STH prevalence data in children disaggregated by age group is shown in Table 2.

**Table 1:** Estimates of Numbers Infected with STHs [90] [Table pntd-0000126-t005]

Parasite	Total Infected (Millions)	Under 5 Years (Millions)
*Ascaris lumbricoides*	1221	122
*Trichuris trichiura*	795	86
Hookworm	740	21

**Table 2:** Published Reports of Prevalence of STH Infections in PSAC Population

Country	Sample Size	Age Range (Years)	Prevalence (%)			Reference
			*Ascaris*	*Trichuris*	Hookworm	
Myanmar	1,206	2–12	81	5	2	[63]
Bangladesh	1,402	2–6	71	44	10	[72]
Indonesia	280	2–5	55	29	0	[91]
Mexico	508	2–10	NS	100	0	[73]
China	329	0–4	66	34	24	[92]
Philippines	544	0–3	3^a^, 19^b^	1^a^, 35^b^	NS	[93]
DRCongo (formerly Zaire)	100	0.5–2	66	48	7	[94]
Tanzania (Zanzibar)	467	0.5–6	40	68	51	[13]
India	1,061	1.5–3.4	50	NS	NS	[65]
Kenya	460	0.5–6	20	15	29	[12]
Brazil	200	2–6	35	13	NS	[95]
Ethiopia	7,155	1–4	38	54	10	[96]
Malaysia	272	3–6	20	24	NS	[97]
Madagascar	864	0–10	88	43	19	[98]
South Africa	200	4–6	82^c^, 81^d^	96^c^, 57^d^	44^c^, 4^d^	[88]
Ghana	422	1–5	62^c^, 23^d^	40^c^, 2^d^	18^c^, 13^d^	[99]
Nigeria	689	1–5	39	36	43	[100]

a Urban area.

b Rural area.

c Coastal area.

d Inland area.

NS, not specified.

The first years of a child's life are marked by intense physical and mental development. In resource-poor settings, this development is compromised by a number of factors, including worm infections that exacerbate already high levels of anaemia and wasting malnutrition. Below are some of the most common health indicators affected by chronic STH infections: iron status, nutrition and growth, vitamin A status, and cognitive development.

### Iron Status

In PSAC whose iron stores are already depleted by malnutrition, additional losses due to hookworms (the most pathogenic of the three STHs), even at low intensities of infection, make a significant contribution to further lowering their haemoglobin levels and triggering anaemia.

Studies in East African PSAC clearly demonstrated a strong correlation between hookworm infection and anaemia. In a community in coastal Kenya, severe anaemia was associated with hookworm infections (>200 eggs per gram) at all ages (range 6–76 months), in both sexes, and independently of socioeconomic factors [12]. In Zanzibar, Tanzania, low haemoglobin concentrations were associated with malaria parasitaemia in children <30 months, and with hookworm intensity in children aged 30–71 months. Importantly, this study also found an association between intensity of hookworm infection and other iron deficiency indicators, such as serum ferritin and erythrocyte protoporphyrin [13]. Theoretical calculations on the quantity of blood lost through hookworm infections also concluded that the parasite could be a leading cause of iron deficiency in PSAC [14].

With regard to *T. trichiura*, moderate-heavy infections have similarly been associated with higher anaemia levels, particularly in malnourished children [15], and co-infection with *T. trichiura* and hookworms may exacerbate hookworm-mediated anaemia, although this has only been studied in SAC to date [16].

There is no evidence that *A. lumbricoides* infection leads to iron malabsorption and iron deficiency anaemia in children.

### Nutrition and Growth

There is now a substantial body of research that clearly demonstrates how STH infections impair healthy nutrition [3],[5]. Growth in STH-infected children is compromised through a variety of mechanisms, including reduced food intake due to malabsorption and/or reduced appetite [5]. As a result, infected children show higher levels of stunting [3],[14],[17]. A study in northeast Brazil showed that in a cohort of children aged 2–7 years, helminthiasis acquired in early childhood was associated with a 4.6-cm shortfall at the age of 7 [18]. Even light infections may have a deleterious effect on protein metabolism, appetite, and erythropoiesis [17]; such effects may be mediated by cytokines (IL 1, IL 6) that are produced by the body's immune response to new helminth infections in naïve children.

### Vitamin A Status

Ascariasis (and trichuriasis in some cases) is associated with low serum vitamin A (retinol) levels through different mechanisms [19]. *Ascaris* lives in the gut, where it interferes with the absorption of vitamin A [20]. In PSAC whose diet is already dangerously low in vitamin A, *Ascaris* infection may trigger clinical vitamin A deficiency and thus may significantly contribute to increased morbidity (such as blindness) and mortality. A study in Nepal found that prevalence of xerophthalmia—the ocular manifestation of vitamin A deficiency—was three times higher in *Ascaris*-infected children aged 6–120 months than in an uninfected group [21].

### Cognitive Development

For obvious reasons, the negative association between helminth infections and mental performance has mainly been studied in SAC [22],[23]. Yet between birth and entering school, significant skills are acquired in different domains that pave the road for the rapid cognitive development that takes place at around 6 years. Although only one large-scale randomised study has been carried out in children aged 6–59 months [24], it is evident that STH infections negatively impact motor and language development. STH infections early in life may therefore negatively affect these cognitive indicators when they are measured later in life [24],[25]. Children between 17 and 72 months of age affected by *Trichuris* Dysentery Syndrome (TDS), a severe form of trichuriasis, showed a marked decrease in cognitive score tests compared with matched controls [26]. This could be due to a variety of causes, both direct and indirect, including induced sleeplessness and micronutrient losses, mainly iron. Changes in cognitive performance may also be plausibly linked with inflammatory response and cytokines triggered by parasitic infections, although this is still under study [27].

### Acute Consequences

In young children, acute consequences of STH infections should not be overlooked. Intestinal obstruction is one of the life-threatening consequences of *Ascaris* infections and is most frequent in children <10 years of age. It is estimated that 12 million cases occur annually worldwide with approximately 10,000 deaths. The incidence reported by published studies is between 0 and 0.25 cases per year per 1,000 population in endemic areas, with a mean case fatality rate of >5% [28]. TDS, already mentioned above, is another acute complication associated with heavy *Trichuris* infection; it mainly occurs in children and leads to severe growth stunting and cognitive deficits that might not be reversible despite treatment [26].

### Interactions with Other Infections

Several field-based observational studies have suggested that STH infections increase the risk of infection with—and adversely affect the clinical outcome of—concomitant malaria, tuberculosis, or HIV/AIDS. Several possible explanatory mechanisms have been put forward, mainly based on an altered immunological response to the infection (and to vaccination in the case of tuberculosis) in individuals who are already infected with STHs. To date, most of this research has been carried out in older age groups with contrasting results. However, the available evidence suggests that treating PSAC for STHs may act as a preventive and possibly a co-therapeutic measure for these three infections. Further research is needed to elucidate the complex relationship between STHs and other infections in co-endemic settings [29]–[33].

## Use of Anthelminthics in Children under 5

### Safety

To date, millions of children—including PSAC—have been routinely administered one or more of the four anthelminthic drugs recommended for STH treatment: albendazole, mebendazole, levamisole, and pyrantel. In 2005, about 48 million PSAC were administered at least one dose of any of the four drugs (WHO, unpublished data); in 2004, slightly less than 37 million were covered [2].

#### Albendazole

At the dosage recommended for STH treatment, the incidence of side effects following treatment reported in the literature is very low. Examples include migration of *A. lumbricoides* through the mouth, occasional gastrointestinal symptoms (epigastric pain 0.3%, diarrhoea 0.3%, nausea 0.2%, vomiting 0.1%), central nervous system symptoms (headache 0.2%, dizziness 0.1%), and rare allergic phenomena (oedema 0.7/1000, rashes 0.2/1000, urticaria 0.1/1000). All these reactions are minor and transient, usually spontaneously disappearing within 48 hours from onset without need for hospitalisation [34],[35].

#### Mebendazole

A few instances of erratic migration of *A. lumbricoides*, mild gastrointestinal disturbances, transient abdominal pain, and diarrhoea have been reported following treatment with mebendazole [34]. Several studies that examined the use of benzimidazoles in children under 24 months reported no incidence of serious adverse experiences, and side effects (either reported or actively searched for) were negligible [4]. The most recent evidence comes from a placebo-randomised trial where there was no increase in the incidence of any side effects in children (aged 6–59 months) treated with mebendazole (500 mg) compared to the control group [36].

#### Levamisole

No literature was found specifically on the use of levamisole in PSAC; however, levamisole has been used for mass administration in China, Iran, Vietnam, Brazil, Kenya, and Nigeria. Reported adverse reactions include occasional vomiting (5%), dizziness (3%), headache (3%), and weakness (2%); all such reactions were mild and transient [37]–[39]. No abnormalities in haematological laboratory tests have been detected with a single dose calculated on the basis of weight (2.5 mg/kg). There have been, however, reports of an increased but low risk of demielizing encephalitis in a large-scale study in China following treatment with levamisole [40]. Also, in Vietnam, STH treatment with levamisole was associated with severe central nervous system disorders in 116 cases who were treated with a locally manufactured generic form of levamisole (Centre for Adverse Drug Reaction, Ministry of Health, Vietnam, unpublished report). There are reports of some drug interactions when levamisole is co-administered with albendazole or ivermectin [41].

#### Pyrantel

Pyrantel has been extensively used in numerous helminth control programmes, particularly in Southeast Asia and Latin America. In a study of 1,506 individuals including children under 5 years of age, side effects were mild and transient and included occasional diarrhoea (4.3%), abdominal pain (4%), nausea (3.5%), vomiting (2%), and headache (3%) [42],[43]. Temporarily raised serum transaminase was detected in 2% of patients.

#### Safe administration

Given the findings that these drugs are extremely well tolerated by infected and non-infected individuals and whole communities at risk of STH infections, WHO recommended that it is safe for paramedical and trained non-medical personnel to administer the drugs. However, it should be noted that while the drugs themselves are extremely safe, their *administration* must also be safe. This is particularly relevant in campaign settings where hundreds of young children (usually PSAC) pass through the health posts each day and multiple products are being administered. Following reports that some of the youngest children were having difficulty swallowing the relatively large deworming tablet, WHO carried out operational research in 2006 to assess the problem. Data from Madagascar and Rwanda found that 1% and 0.1%, respectively, of the PSAC choked on the tablet (WHO, unpublished data), which prompted concerns and interim recommendations from WHO, including the need for appropriate training when treating this age group and for the tablet to be broken and crushed with water before administration to children aged 1–3 years [8]; older children should be asked to chew the tablets in front of the administrator and children should never be forced to swallow the tablet, which can cause choking or asphyxiation (UNICEF Ethiopia, unpublished report); and health posts must be set up logically with the products delivered in a specific order—WHO recommends that the vaccine is given after the vitamin A and anthelminthic, and a crying child should never be forced to swallow the tablet [8].

In some countries, suspension formulations are used to treat PSAC. The drawback is that syrups are more expensive and more difficult to store and transport [9].

### Safety of Co-Administration

Anthelminthic drugs against STH are now routinely delivered with other anthelminthics, with vitamin A supplements, and with certain vaccines. Although pharmacokinetics of anthelminthics has been extensively investigated [44], no specific studies have evaluated the pharmacokinetic and clinical drug interactions of STH anthelminthics with other products when co-administered to either SAC or PSAC, except for albendazole with praziquantel or DEC; and pyrantel with oxantel.

#### Albendazole+diethylcarbamazin

Albendazole and diethylcarbamazin (DEC) for the treatment of lymphatic filariasis can be administered to children from their 2nd birthday onwards [9]. No increase in the number of adverse reactions has been reported as compared to each drug being delivered alone [45]. As of 2002, the monitoring and evaluation system of the global programme to eliminate lymphatic filariasis has reported that 5.3% of those administered with albendazole+DEC (all age groups) had experienced an adverse reaction of a gravity sufficient to prevent normal daily activities [46].

#### Albendazole+ivermectin

The use of this combination for the treatment of lymphatic filariasis is restricted to individuals ≥15 kg in weight or ≥90 cm in height [9]. It has been shown that in countries where height is used as the selection criterion, a large proportion of PSAC is included in the target population because the threshold height of the treatment pole (90 cm) often selects children 5 years old and younger [47]. The monitoring and evaluation system of the global programme to eliminate lymphatic filariasis reported that as of 2002 a small proportion (1.5%) of those administered with albendazole+ivermectin (IVM) (all age groups) had experienced an adverse reaction of a gravity sufficient to prevent normal daily activities [46].

#### Pyrantel+oxantel

Pyrantel given with oxantel has proved to be more effective against STHs than either drug alone [48],[49], particularly against *T. trichiura* [50]. No serious adverse experiences have been reported in large-scale helminth control programmes [51].

#### Albendazole/mebendazole+vitamin A

WHO recommends that any of the two benzimidazoles above can be safely co-administered with vitamin A supplements [11]. There are no published epidemiological studies investigating the pharmacological interaction or the clinical safety of co-administration of these two products. However, after multiple years where these two interventions have been co-delivered in countries across the world, the authors are not aware of any increased frequency of adverse reactions or serious adverse experiences.

#### Anthelminthic drugs+vaccinations

As previously mentioned, there is evidence that helminth infections modulate the immune response to unrelated pathogens, and they have been implicated in the poor efficacy of some vaccines, either currently used, for example those against tuberculosis (BCG) or cholera, or experimental, such as that against malaria [32],[52],[53]. Conversely, deworming treatment before antimalarial immunization restored the protective immunity to malaria challenge in nematode-infected mice [53], showing that anthelminthic treatment might boost immunological response in infected children. Although there is no policy regarding co-administration of anthelminthic drugs and vaccinations, several large-scale interventions currently deliver albendazole or mebendazole with measles, polio, or BCG vaccines. The authors are at present not aware of an increased frequency of adverse reactions or serious adverse experiences following implementation of such interventions.

## Drug Efficacy—Reducing the Worm Burden

The broad spectrum efficacy of both benzimidazoles, levamisole, and pyrantel in reducing the prevalence and intensity of STH infections has been extensively described [54],[55]. Drug efficacy varies according to a number of variables, including the dose, the helminth species the parasite strains involved, the transmission and intensity of infection, the diagnostic method used, and the timing of assessing efficacy after treatment [56]. However, drug efficacy is not influenced by age, and therefore it is assumed that results from efficacy studies in age groups other than PSAC are also applicable to this age group.

- Formulation and dosages [57],[58].
  - All four anthelminthics are given as a single dose and are not recommended in children <12 months.
  - Albendazole (chewable and non-chewable tablets 200 and 400 mg; oral suspension 100 mg/5 ml, 200 mg/5 ml) is given at the dosage of 400 mg for all ages (except for children between 12 and 24 months. who receive 200 mg).
  - Mebendazole (chewable and non-chewable tablets 100 and 500 mg; oral suspension 100 mg/5 ml) is given at a dosage of 500 mg for all ages.
  - Levamisole (chewable tablets 40, 50, and 150 mg; syrup 40 mg/5 ml) is given at the dosage of 2.5 mg/kg.
  - Pyrantel (chewable tablets 250 mg; oral suspension 50 mg/ml) is given at the dosage of 10 mg/kg.
- All four anthelminthics are very effective against *A. lumbricoides*, with cure rates (CRs) and egg reduction rates (ERRs) between 90% and 100%.
- Albendazole is effective against the hookworms (CR 57%–95% and ERR 79%–99%) and has been shown to be more active than other anthelminthics against *N. americanus.*
- Mebendazole is less effective in curing hookworms than albendazole, but nevertheless reduces the worm burden by >80%.
- Levamisole and pyrantel are active against hookworm infections, although benzimidazoles are the drug of choice in large scale deworming campaigns. Levamisole is less effective in curing infections, and both drugs have been shown to be more effective against *A. duodenale* (ERR>80%) than against *N. americanus*, where repeated doses are needed, especially in heavy infections.
- Both benzimidazoles (albendazole and mebendazole) have similarly poor efficacy in curing *T. trichiura* infections (CR between 10% and 77%), but significantly reduce the worm burden (ERR 60%–80%).
- Levamisole and pyrantel have little effect on *T. trichiura*.

It is essential to recognise that in endemic areas, re-infection is the rule and the objective of regular deworming is not to cure since re-infection is inevitable, but to reduce and keep the worm burden of infected individuals below the threshold that is associated with morbidity [59]. There is the threat that after several anthelminthic treatments, drug efficacy may be impaired due to selection of resistant parasites. Anthelminthic resistance is not yet a public health problem in human STHs; nevertheless, drug efficacy should be monitored where large-scale deworming programmes are in place [60]. In 2006, a study to investigate the efficacy of albendazole in PSAC was carried out in Nepal, where the drug has been regularly delivered over the past 5 years. No sign of reduced efficacy due to drug resistance was found (Marco Albonico, personal communication).

## The Health Impact of Regular STH Deworming in Endemic Areas

Periodic STH deworming has been shown to improve growth, micronutrient status (iron and vitamin A), and motor and language development in PSAC, and makes a strong case for including this age group in control programmes where STH helminth infections are prevalent. Below we describe the effectiveness of the drugs in reducing morbidity. It is worth noting that some of the trials described included a passive or active pharmacovigilance component, which further confirms the safety of administering anthelminthic drugs to this age group.

### Impact on Morbidity

Unlike some other parasitic infections, morbidity attributable to STHs is often subtle and non-specific, and therefore difficult to assess. Typical symptoms include vomiting, coughing, and abdominal pain. In some cases, more specific signs such as passing worms are observed. The frequency of all these symptoms in PSAC and SAC infected with *A. lumbricoides* in rural Myanmar was drastically reduced by 6-monthly treatment with levamisole [61].

Taking each of the morbidity indicators most associated with STH infections in turn:

#### Growth

A summary of published studies that demonstrate the improvement in growth of PSAC after anthelminthic treatment is shown in Table 3.

**Table 3:** Impact of Anthelminthic Treatment on Growth of PSAC

Country	Sample Size	Age Range	STH	Treatment Schedule	Follow-Up (Months)	Outcome	Reference
Myanmar	1,206	2–12	A T H	LEV/3 months	24	0.93 kg more in weight, 0.6 cm more in height compared with no treatment	[63]
Bangladesh	1,402	2–6	A T H	MBD 500 mg/2 months	18	No improvement in weight, height, or mid-arm circumference compared with placebo.	[72]
Indonesia	289	2–5	A T	ALB 400 mg+iron supplement	2	0.17 kg more in weight and no difference in height compared with placebo.	[91]
Mexico	508	2–10	T	ALB 400 mg or PYR 11 mg/kg/4 months	12	After ALB 0.01 kg less in weight and 0.14 cm more in height than after PYR.	[73]
Tanzania	273	1–5	A	LEV/3 months	12	Weight gain 21% greater than that in those given a placebo.	[62]
Tanzania (Zanzibar)	459	0.5–6	A T H	MBD 500 mg/3 months	12	Mild wasting malnutrition reduced by 62% and small arm circumference by 71% in children <30 months. Appetite improved by 48% in all children.	[17]
Uganda	28,015	0–7	A T H	ALB 400 mg/6 months	30	154 g weight gains of the treated group compared with no treatment.	[64]
India	1,061	1.5–3.5	A	ALB 400 mg/6 months	24	9.4% less stunting compared with placebo.	[65]
India	702	2–5	A	ALB/6 months	12	40% ALB treated children had >10% weight gain compared with 29% of placebo-treated children.28% reduction in diarrohea episodes.	[68]
Kenya	574	2–4	A T H	MBD 500 mg	6	0.44 kg weight gain, 0.78 cm height gain, 0.28 weight for age (Z score) gain, compared with placebo.	[70]
DR Congo (formerly Zaire)	328	0–6	A T	MBD 500 mg/3 months	12	No gain in weight nor in height compared with no treatment.	[74]

A, *A. lumbricoides*; ALB, albendazole; H, hookworm; LEV, levamisole; MBD, mebendazole; T, *T. trichiura*.

In Tanzania, PSAC had a demonstrable growth benefit when they received repeated treatment with levamisole for their *Ascaris* infections [62].

In Myanmar, 3-monthly levamisole treatment resulted in greater weight (0.93 kg) and height (0.6 cm) gain in treated children (age 2–12 years) when compared to an untreated group at the 24-month follow-up. The results were also statistically significant when the 2- to 5-year age group was analysed separately [63].

In Zanzibar, children aged 6–59 months of age with a heavy burden of helminth infections and malnutrition were studied during a placebo-randomised trial to measure the effect of a daily low-dose iron and/or 3-monthly STH treatment on growth, iron status, anaemia, and development. At 12 months, the results found that periodic mebendazole had reduced mild wasting malnutrition by 62% and small arm circumference by 71% in children aged <30 months. In all children, their appetite had improved by 48% [17].

In Uganda, a randomised trial showed the weight of PSAC who received albendazole every 6 months during Child Health Days was 10% greater than in untreated controls (5% greater when the treatment was given annually) [64].

In a series of studies in Indian urban slums, the delivery of albendazole every 6 months reduced stunting by 9.4% and improved weight by 35% in infants and PSAC within 2 years [65],[66]. A large-scale study then confirmed these findings [67], and a significant weight gain and a 28% reduction in diarrhoea episodes in PSAC were further observed following treatment with albendazole [68].

In Kenya, treatment of sick, STH infected 2- to 4-year-olds with a single dose of mebendazole through the Integrated Management of Childhood Illness (IMCI) approach yielded statistically significant height and weight gains at follow up after 6 months [69],[70]. The growth of *Ascaris*-infected PSAC in Kenya also benefited from repeated levamisole treatments [71].

A few studies have failed to show any impact of deworming on growth:

- A study from Bangladesh in children 2–6 years old who were treated with mebendazole every 2 months did not show improvement in weight, height, or mid arm circumference when compared to the placebo group [72]. A high prevalence of *Giardia duodenalis* in the treated group might account for the poor outcomes.
- A trial in Mexico that examined the effects of albendazole (400 mg or 1,200 mg) or pyrantel treatment on growth of apparently healthy children (aged 2–10 years) infected with *T. trichiura* did not show any significant difference in growth at a 12-month follow-up between the three drug regimens [73].
- In the Democratic Republic of the Congo (formerly Zaire), deworming with mebendazole every 3 months did not improve the growth of malnourished children aged 0–72 months, whereas vitamin A supplements did have an effect at a 12-month follow-up. In this study, however, the prevalence of STH infections was low (10% *A. lumbricoides* at baseline) and the manufacturer of the mebendazole was not mentioned [74].

#### Iron status

A summary of published studies on the improvement in iron status in PSAC following STH deworming is shown in Table 4.

**Table 4:** Impact of Anthelminthic Treatment on Iron Status and Vitamin A of PSAC

Country	Sample Size	Age Range	STH	Treatment Schedule	Follow-Up (Months)	Outcome	Reference
Tanzania (Zanzibar)	459	0.5–6	A T H	MBD 500 mg/3 months	12	Moderate anaemia (Hb <9 g/dl) reduced by 59% in children aged <24 months compared with placebo.	[17]
Nepal	1,635	2–5	A T H	ALB 400 mg/6 months+vitamin A	12	Anaemia reduced by 77%. No control group.	[75]
Indonesia	259	3–6	A	LEV+beta carotene+fat meal	1	Serum retinol increased in Lev group compared with beta carotene+fat meal.	[76]
Indonesia	131	2.5–5	A T	ALB+vitamin A	1	Vitamin A deficiency improved by 81% and anaemia reduced by 39%.	[77]
India	487	1–5	A	L-tetramisole+vitamin A	12	Deworming did not augment the beneficial effect of vitamin A. Increment of vitamin A at follow up in the ascaris +ves was 65% of that of ascaris −ves.	[78]

A, *A. lumbricoides*; ALB, albendazole; H, hookworm; MBD, mebendazole; T, *T. trichiura*.

In Zanzibar, the administration of mebendazole to children <24 months every 3 months was shown to reduce moderate anaemia (Hb<9 g/dl) by 59% after 12 months [17]. Preliminary reports from a large scale trial (*n* = 2,000 children aged 6–23 months) carried out in 2005 in the same study area suggest a significant decrease of wasting malnutrition and of moderate-severe anaemia in the mebendazole treated group (Rebecca Stoltzfus, personal communication).

In Nepal, a study in 2004 to evaluate the national biannual vitamin A supplementation programme that has co-delivered STH treatment since 1999 demonstrated an impressive drop in anaemia (from 47% to 11%) in children aged 12–59 months [75]. This profound benefit could be attributed to the control of hookworm infections and to the parallel delivery of vitamin A supplements, as both interventions reduce anaemia.

Except for the two trials mentioned above, there are no other studies that have examined the impact of regular anthelminthic treatment on the iron status of PSAC living in hookworm-endemic areas.

#### Vitamin A

A summary of published studies on the improvement of vitamin A status in PSAC following STH deworming is shown in Table 4.

In Indonesia, two studies examined the impact of deworming on vitamin A status. In the first study, fatty meals and beta-carotene supplements were given to 3- to 6-year-old children infected with STHs. A subgroup of these children was also dewormed with levamisole. Dewormed children showed the highest rise in serum retinol. Moreover, additional meal fat combined with STH deworming increased serum retinol to the same degree as feeding the children with additional beta carotene sources [76]. In the second study, the vitamin A was given just before the deworming; again, children cleared of *A. lumbricoides* or *T. trichiura* infections showed a significantly improved vitamin A status at follow up [77].

In urban slums in Hyderabad, India, where *A. lumbricoides* is endemic, a randomised study in 1- to 5-year-old children indicated that deworming with L-tetramisole did not augment the beneficial effects of vitamin A, and that deworming alone did not improve their vitamin A status as compared to the placebo group. At the 1-year follow-up, however, the vitamin A levels in the *Ascaris*-infected group showed less improvement than those in the uninfected group, although the results were not statistically significant [78].

#### Cognitive and motor development

Only a few studies have evaluated the impact of periodic deworming on cognitive and motor development of PSAC due do the difficulties of designing a well-controlled study and applying cognitive tests and measuring standard development milestones in this age group and in different socio-cultural settings.

In Zanzibar, periodic treatment of STH-infected PSAC (*n* = 417) with mebendazole had a positive, although not statistically significant, effect on children's motor and language development; most of the effect was observed in children between 12 and 24 months of age [24].

In a Caribbean setting, repeated treatment with mebendazole improved the mental development of trichuris-infected PSAC aged 27–72 months suffering from TDS [79].

In India, a study of children aged 18–42 months (*n* = 1,016) who were divided into a treatment and control group showed no difference in their cognitive performance following treatment with albendazole twice yearly at a 24-month follow-up [65].

## Experience from the Field: Scaling up Deworming by Incorporating STH Control into Other Large-Scale Public Health Interventions

The number of PSAC being dewormed is rapidly escalating due to the fact that countries are adding deworming to established large-scale programmes, including vitamin A supplementation programmes, vaccination campaigns, and Child Health Days. The advantage of these programmes is that they are often delivered regularly, the anthelminthic drugs are safe (it is difficult to overdose on them), and with proper training, their administration is also straightforward (unlike injections, for example). For the latter two reasons, anthelminthics can be given by trained non-health staff. Co-delivery using an existing infrastructure also reduces costs, improves access to difficult-to-reach communities, and boosts overall coverage since a package of medications is usually more appealing than a single one [6].

Although WHO's reporting system has strengthened over recent years, which partly explains the increase in numbers, WHO estimates that 10 million PSAC were reached in 2003, 37 million in 2004, and nearly 50 million in 2005 [2]. Country reports and data are collected by WHO and available on the Web [80].

### Delivery Channels

#### Vitamin A campaigns

The co-delivery of vitamin A capsules and STH deworming tablets is a particularly logical union for several reasons. First, the target age group is similar: vitamin A is given to children 6–59 months of age and deworming to 12- to 59-month olds. Second, vitamin A is delivered twice a year, which is the same frequency required for STH deworming of PSAC if the prevalence of infection is ≥50% in the school-age population. Third, vitamin A programmes enjoy a high coverage. In 2004, approximately 190 million children received at least one high-dose vitamin A supplement and global coverage reached 68% of targeted children [81]. Fourth, if a child is vitamin A deficient, he/she is likely to be also infected with worms given that both are common in the same socio-economic conditions. Fifth, *A. lumbricoides* impairs the absorption of vitamin A from the diet, either by competing for vitamin A absorption with the host, or by leading to overall malabsorption of vitamin A (and other micronutrients) in the gut; this is especially true in chronic infections [11],[20].

#### Supplementary immunization activities

Supplementary immunization activities (SIAs) are active vaccination interventions that complement routine immunization activities. Such interventions or campaigns are ongoing in many countries and have extremely well-established delivery systems that target all infants in their first years of life. Several countries have taken advantage of their SIAs and have included STH deworming as part of their measles and/or polio vaccination campaigns [82]. The benefit of using these campaigns (there are several types, including catch-up, follow-up, and mop-up campaigns for measles, and national and sub-national immunization days [NIDs and sub-NIDs] for measles and polio) for delivering other products is that they have an extensive coverage. Target groups for deworming and vaccines are not the same but largely overlap: measles vaccine is given from 9 months of age onwards (up to 59 months usually). Polio targets 0- to 59-month-old children. The drawback is that STH deworming must be delivered regularly, while measles campaigns only take place every 3–4 years; polio NIDs or sub-NIDs are usually more frequent, but less regular depending on epidemiology of infection. Also, while coupling deworming tablets with the oral polio vaccine does not pose significant logistical problems to the community volunteers responsible for its administration, some countries have voiced concerns that deworming added to measles campaigns may jeopardize the campaign (the measles vaccine is injected only by trained medical personnel, which makes the burden of work per person high).

#### Child Health Days

Child Health Days and Child Health Weeks are regular and systematic interventions aimed at improving access to, and use of, routinely available health services [82]. They are becoming increasingly popular as a way of reaching large numbers of children under five years old as well as SAC (and sometimes adults) in many countries. In Uganda, for example, different baskets of products and services are co-delivered annually or biannually, including vaccines, vitamin A supplements, deworming tablets, and insecticide-treated nets, plus a range of services including growth monitoring, antenatal care, and health education and family planning, according to the needs of the country [83].

#### Routine health delivery services

Routine health delivery services are interventions taking places at health centres all year round. Both EPI (Expanded Program on Immunization) and IMCI (Integrated Management of Childhood Illness) are examples of routine services. EPI provides routine vaccinations according to the national immunization schedule. Immunization carried out at health centres can be complemented by outreach activities whereby mobile vaccinators regularly (usually every month) visit dispersed communities so as to increase vaccination coverage. Even if EPI mainly targets children in their first year of life, in many developing countries a significant proportion of children older than 1 year still have to complete their vaccination schedule. In this circumstance, the EPI visit represents a good opportunity for providing STH deworming tablets.

The IMCI strategy aims to improve the routine clinical management of PSAC; its approach includes the accurate identification and management of illnesses in health facilities and represents another delivery channel to reach PSAC. Treatment of STH infection is included in the IMCI protocol: administration of mebendazole treatment is recommended for sick children older than 2 years with palmar pallor or with very low weight for age in areas where hookworm and *T. trichiura* infections are endemic. Successful examples of deworming through IMCI are reported from Kenya and Mexico [69],[70],[84].

#### Schools and nurseries

Schools offer an excellent opportunity for reaching schoolchildren, with the advantage that schoolteachers can be trained to distribute anthelminthic drugs to their pupils. However, schools can also represent an entry point into pupils' families and the wider community. Experience from Zanzibar showed that both PSAC and non-enrolled SAC can be successfully reached through the local school deworming programme [85]. It is worth noting that in many African countries late school enrolment is common and children often start primary school at the age of 8–9 years old [86]. Where school enrolment or the number of schools is very low. school-based distribution needs to be coupled with other delivery channels. In some countries, like the Seychelles [87] and South Africa [88], crèches and nurseries have been successfully used to reach PSAC.

#### Community-based anthelminthic interventions

These are interventions utilizing community members as distributors of anthelminthic drugs. Such interventions are commonly implemented when entire communities—and not specific population groups—are targeted for treatment, such as in the case of onchocerciasis control (community-directed treatment with ivermectin [CDTI]) and lymphatic filariasis elimination (mass drug administration [MDA], with either ivermectin+albendazole or DEC+albendazole). According to the drug(s) being delivered, PSAC (defined slightly differently according to the programme in question) are included among those eligible for treatment [9]. Under the lymphatic filariasis programme, a child, by default, is covered for STH infection through the albendazole component of the drug combination. Whereas under the onchocerciasis programme, which only delivers ivermectin (ivermectin is not recommended for STH control due to its suboptimal effect against the STH parasites [9]), the simultaneous distribution of albendazole for STH deworming could be considered, which would take advantage of the established onchocerciasis delivery channel. The drawback of community-based interventions for both lymphatic filariasis and onchocerciasis is that they usually take place once a year, which may not be sufficient to effectively control STH infection in high-risk communities where two yearly distributions are recommended [9]. Where school enrolment is low, community-based distributors are also used to treat SAC and other eligible children who are not yet at school with other anthelminthics such as praziquantel [89].

## Conclusion: Need for Further Science-Based Evidence

Periodic treatment with anthelminthics has become one of the most popular and successful public health interventions taking place in STH-endemic countries. This paper has endeavoured to summarize the published evidence and country experiences that support and justify the inclusion of pre-school children in these programmes. In conclusion, both sources of information support the recommendation that PSAC should indeed be regularly dewormed where the epidemiological situation merits such action. The published literature clearly shows that PSAC infected with STHs benefit both in terms of their health and development and also in terms of reaching their cognitive potential. Country experiences demonstrate that the addition of deworming to large-scale public health programmes is not only feasible, but also makes sense on a number of practical fronts. However, this trend also raises a number of important issues that should be addressed by the scientific community: (i) monitoring safe administration of the deworming tablets in children 1–3 years old needs to be strengthened; (ii) more appropriate paediatric formulations need serious research and development; (iii) stronger economic analysis and evidence to assess the cost-effectiveness of deworming PSAC is needed; (iv) more research on the interaction of anthelminthics with vaccines is needed; and (v) more research on how STH infections impact the health of a person co-infected with tuberculosis, malaria, and HIV is required. Some open research questions and operational challenges that would need further consideration in the public health agenda are described in Table 5.

**Table 5 pntd-0000126-t005:** Research and Operational Challenges

**Further assessment of the risk/benefits of deworming PSAC for STH**	**Iron status:** The impact of deworming on the iron status of PSAC needs to be further documented and stronger evidence should be published from large-scale studies.
	**Cognitive development:** The impact of periodic STH treatment on the mental, motor and language development of PSAC needs to be confirmed and properly tested in different epidemiological settings.
**Outreach/cost/feasibility**	**Innovative ways of outreaching and delivering STH deworming to PSAC**—alone or with other medications—should be documented, with particular attention to feasibility and cost-effectiveness. It is essential that such approaches are properly monitored and evaluated at country level, and their successes documented in order to enhance advocacy and guide international agencies and policy makers to promote appropriate tools for the control of STH infections and other diseases in children.
**Monitoring safety**	**1- to 3–year-old children and tablet size:** Adverse Event Reporting Forms (AERS) need to be included in any campaign delivering anthelminthic drugs so as to routinely capture data on the number of children having problems with swallowing tablets. In the meantime, WHO and UNICEF should continue advocating for the development of novel paediatric formulations (rapid dissolving tablets).
	**Pharmacovigilance:** Safety of giving anthelminthics with other drugs in population-based interventions needs to be evaluated in large-scale control programmes by a review of observational evidence.
**Monitoring drug efficacy**	**Drug efficacy** of anthelminthic drugs delivered in large-scale interventions and the early occurrence of treatment failures needs to be monitored; any suspect episode of drug resistance should be properly reported.
**Monitoring coverage**	**An integrated monitoring system** to collect and manage data related to the number of children treated (both PSAC and SAC) needs to be developed to report on coverage of different age and high risk groups.
**Evaluating interactions**	**Vaccine interactions:** Possible synergies and interactions related to delivering anthelminthics with different vaccines (measles, BCG, polio) in large-scale vaccination campaigns needs to be further evaluated and properly followed up in the mid and long term.
	**Malaria/TB/HIV:** Interactions of STH infections with the management of the three big killers—malaria, TB, and HIV—needs further study and science-based evidence to advocate and suggest novel means of combating malaria, TB, and HIV infections in STH-endemic areas.

**Box 1. Search Strategy and Selection Criteria**

Most information for this Review comes from published literature. References were identified through Medline searches between 1969 and 2007. The general search terms used were “helminths” and “children”. The following specific search terms were then used: “helminths and anaemia”, “helminths and vitamin A”, “helminths and malnutrition”, “helminths and malaria”, “helminths and HIV”, “helminths and TB”, and “helminths and vaccine”. Other information was sourced from relevant documents published by WHO, Geneva. Some information, particularly relating to country experiences, was extracted from grey literature and from unpublished country reports in the WHO Global Databank on schistosomiasis and STH.

**Box 2. Learning Points**

1. More than 200 million pre-school-age (PSAC) children are infected by soil-transmitted helminths (STHs), which affects their iron and vitamin A status, and impairs their nutrition, growth, and cognitive development.
2. Safety tested and effective anthelminthic drugs with a significant impact on worm burden and disease-associated morbidity are available for administration to children over 1 year of age.
3. PSAC infected by STHs benefit from preventive treatment with anthelminthics, and country experiences demonstrate that almost 50 million PSAC worldwide have been dewormed for STHs in 2006 through large-scale interventions such as vitamin A and immunization campaigns, Child Health Days, routine health delivery services, schools, nurseries, and community-based interventions.
4. A number of open research questions and operational challenges still need to be addressed, such as monitoring the safety of tablet administration in children 1–3 years old and advocacy for new paediatric formulations, further assessment of the cost-effectiveness of deworming PSAC, and interactions with vaccines and with co-infection with tuberculosis, malaria, and HIV.

**Box 3. Key Papers**

1. Albonico M, Crompton DW, Savioli L (1999) Control strategies for human intestinal nematode infections. Adv Parasitol 42: 277–341.
2. Stoltzfus RJ, Chwaya HM, Montresor A, Tielsch JM, Jape Khatib J, Albonico M, Savioli L (2004) Low dose daily iron supplementation improves iron status and appetite but not anemia, whereas quarterly anthelminthic treatment improves growth, appetite and anemia in Zanzibari pre-school children. J Nutr 134: 348–356.
3. Montresor A, Awashti S, Crompton DWT (2003) Use of benzimidazoles in children younger than 24 months for the treatment of soil-transmitted helminthiasis. Acta Trop 86: 223–232.
4. WHO and UNICEF (2004) How to add deworming to vitamin A distribution. Geneva: World Health Organization.
5. WHO (2006) Schistosomiasis and soil-transmitted helminth infections–preliminary estimates of the number of children treated with albendazole or mebendazole. Wkly Epidemiol Rec 16: 145–164.
6. WHO (2007) WHO Global Databank on schistosomiasis and soil-transmitted helminth infections. Geneva: World Health Organization. Available: http://www.who.int/wormcontrol/databank/en/. Accessed 2 March 2008.
